# Reproductive biology of female Nile tilapia *Oreochromis niloticus* (Linnaeus) reared in monoculture and polyculture with African sharptooth catfish *Clarias gariepinus* (Burchell)

**DOI:** 10.1186/s40064-015-1027-2

**Published:** 2015-06-17

**Authors:** A P Shoko, S M Limbu, H D J Mrosso, Y D Mgaya

**Affiliations:** Tanzania Fisheries Research Institute, Institute Headquarters, P. O. Box 9750, Dar es Salaam, Tanzania; Department of Aquatic Sciences and Fisheries, University of Dar es Salaam, P. O. Box 35064, Dar es Salaam, Tanzania; Tanzania Fisheries Research Institute, Mwanza Centre, P. O. Box 475, Mwanza, Tanzania

**Keywords:** Early maturation, Prolific breeding, Tilapia culture systems, Grow-out ponds, Fecundity, Ovary weight

## Abstract

This study was conducted to assess the reproductive biology and early breeding behaviour of female *Oreochromis niloticus* reared in monoculture and polyculture with *Clarias gariepinus* in earthen ponds for 8 months. Results revealed no significant difference in length at first maturity (L_50_) between females reared in monoculture and polyculture systems. Similarly, no significant differences were detected in absolute fecundity, relative fecundity, gonado-somatic index and condition factor between the two culture systems. The absolute fecundity was more strongly correlated with total length and body weight than with ovary weight. The study concluded that early breeding of *O. niloticus* in captivity is not affected by the culture systems used. Therefore, *O. niloticus* production in either system can be improved only through proper pond management techniques.

## Background

Nile tilapia *Oreochromis niloticus* (Linnaeus, 1757) is a worldwide important species in aquaculture because of its fast growth, firm and tasty flesh, resistance against harsh conditions and ease production of fingerlings under captivity (Fryer and Iles 1972; Yi et al. 1996; de Graaf et al. 1999; Gómez-Márquez et al. 2003). However, early maturation and prolific breeding of *O. niloticus* in culture systems, especially earthen ponds is a major problem in tilapia farming (Bardach et al. 1972; McGinty 1985; Suresh and Bhujel 2012). The large numbers of fingerlings produced through reproduction during grow-out consume the feeds and dissolved oxygen intended for the stocked tilapia (de Graaf et al. 1996). Consequently, the growth rate decreases with fewer marketable-size fish. In the wild, *O. niloticus* starts to reproduce at a total length of 20–30 cm (150–250 g) (Lowe-McConnell 1958; Gwahaba 1973). However, under captivity *O. niloticus* reaches sexual maturity at a relatively smaller size of 8–13 cm (Siraj et al. 1983; de Silva and Radampola 1990; Suresh and Bhujel 2012).

Methods adopted to control overpopulation in farmed tilapias have been reviewed by Guerrero (1982) and Mair and Little (1991). These methods include monosex culture through manual sexing and hormonal sex reversal, cage culture, high stocking density, sterilization, intermittent (selective) harvesting and the use of slow maturing tilapia species or strains. However, these methods have their limitations. For example, the use of reproductive inhibitors (sterilization) such as irradiation or chemosterilants are expensive and require hatchery facilities and skilled manpower. The intervention of hormones is also expensive and sometimes difficult to obtain (Jegede 2010). On the other hand, the use of predator-control method such as African sharptooth catfish *Clarias gariepinus* (Burchell, 1822) through polyculture has been considered a safe biological method for controlling unwanted reproduced fingerlings in mixed-sex tilapia population in ponds without affecting the big size tilapia (Abdel-Tawwab 2005). The reproductive biology of female *O. niloticus* reared in monoculture system has been investigated by de Graaf et al. (1999). However, limited attempts have been made to assess the reproductive biology of female *O. niloticus* in polyculture system. This assessment is useful in ensuring the well-being of the cultured fish (Moreau et al. 1986), enabling protection of new recruits (King and Udo 1998).

The objective of the present study was to determine the reproductive biology of female *O. niloticus* with an emphasis on its early breeding behaviour under monoculture and polyculture pond systems. In order to address this objective, it was hypothesised that *O. niloticus* reared in monoculture system attains sexual maturity earlier and at a smaller size than those reared under polyculture system.

## Methods

### Ethical statement

This study was not evaluated by an Animal Ethics Committee because there was no such committee in Tanzania during the course of the study. Nevertheless, the research methods used for fish sampling followed internationally recognised guidelines for ethical use of animals (for example, Håstein 2004; Grigorakis 2010). The fish used for determination of maturity stages were sacrificed by hypothermia through immersing them in an ice-slurry to avoid causing stress and pain before death (Sneddon 2006). By using ice water, it was possible to calm down the fish for several hours until osmoregulatory problems and exhaustion occur. It has been demonstrated that pre-chilling prior to slaughter is a minor stressor (Sneddon 2012).

### Study site

**Figure 1:**
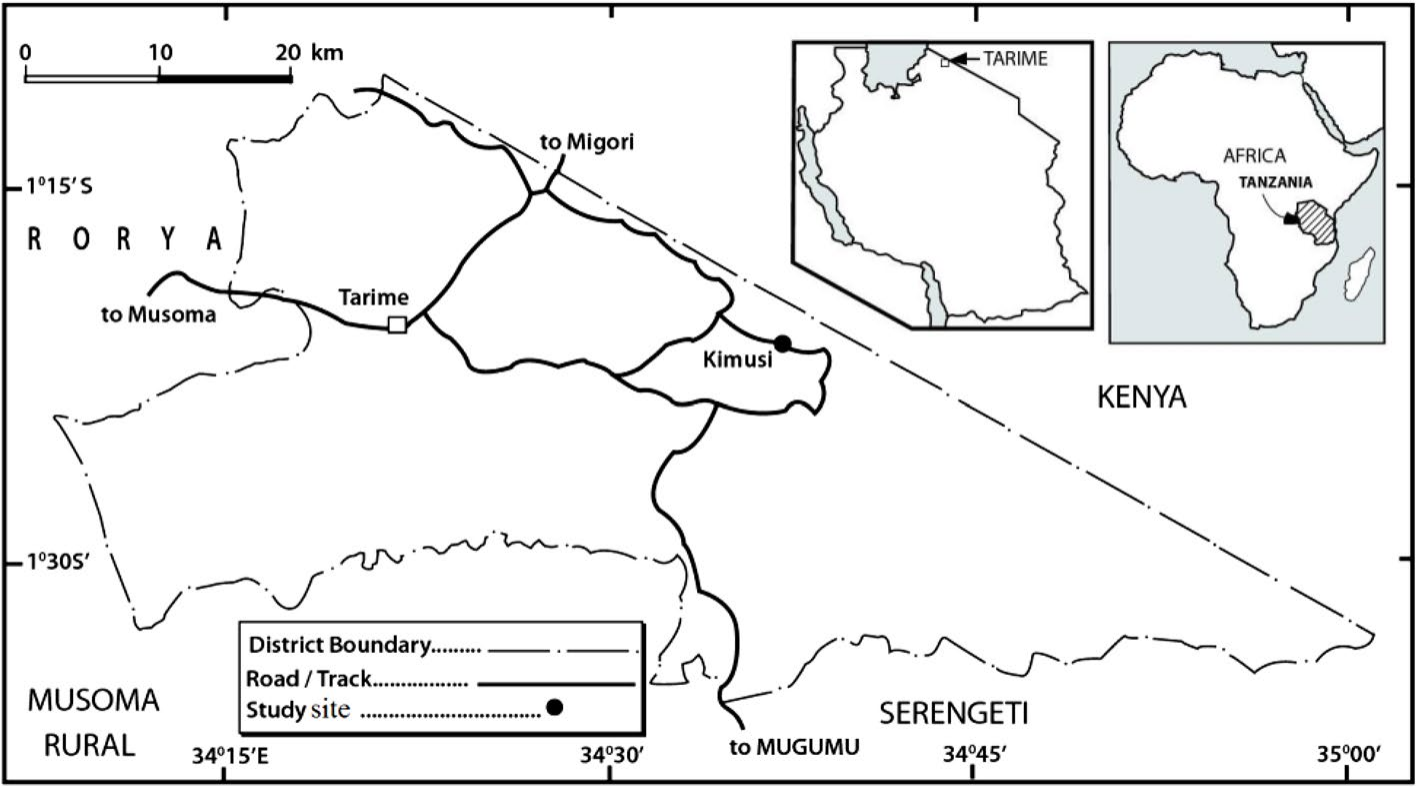
Map of Tarime District, Tanzania showing study site (source: University of Dar es Salaam Cartographic Unit).

The study was conducted in the Lake Victoria basin, Tanzania at Kimusi village, Tarime District, of Mara Region (Figure 1). Kimusi village is located between latitudes 1°10″ and 1°36″ South of the Equator and longitudes 34°08″–35°01″ East of Greenwich Meridian at an altitude ranging from 1,500 to 1,800 m above sea level. The mean annual rainfall in this area ranges from 1,200 to 1,500 mm while the range of mean annual temperature is 18–26°C. This village has deep, well-drained, red or brown soils on the gentle hillsides. In the valleys the area become shallow and stony on the steeper slopes and dark-grey or brown clays with obstructed drainage. The area is characterized by the fertile soils which make it important in agricultural production (URT 1998).

### Experimental procedures

The experiment was conducted in six earthen fish ponds of 150 m^2^ with a mean depth of 1.25 m each. The ponds received water from the source of Mori River that drains to Lake Victoria. Three ponds were stocked with *O. niloticus* and *C. gariepinus* in polyculture while the other three ponds were stocked with *O. niloticus* in monoculture. A stocking ratio of 1:3 for *C. gariepinus* and *O. niloticus* was adopted for polyculture treatment. Both culture systems were stocked with fingerlings of mean (±SE) initial weights of 5.01 ± 0.01 g at a stocking density of 9 fish m^−2^. For polyculture ponds, stocking of similar initial weights of *C. gariepinus* was intentionally delayed for 30 days to allow *O. niloticus* to grow to a size (12.26 ± 9.49 g) that *C. gariepinus* were unable to prey on (Shoko et al. 2014a).

In order to regulate pond water pH and water chemistry in general, before fish stocking, the ponds were drained and limed with dolomite at a rate of 0.25 kg m^−2^ (Engle and Neira 2005). Fish were fed on 297.50 g kg^−1^ crude protein diet made using locally available cotton seed cake 683.40 g kg^−1^ and maize bran 316.60 g kg^−1^ of dry feed (Table 1) adopted from Shoko et al. (2014b). Food was supplied at a constant daily feeding rate of 5% per body weight for *O. niloticus* or for the combined body weights of *O. niloticus* and *C. gariepinus* in the case of polyculture. This feeding rate was done so intentionally to reflect the actual practice by small scale rural fish farmers in the study area. The ration was apportioned into two and fed twice per day in the morning between 09:00 and 10:00 h and evening between 15:00 and 16:00 h. Fish sampling which was done on a monthly basis started during the third month of the experiment to allow for gonadal development. The duration of the study was 8 months.

**Table 1:** Formulation and proximate composition (dry matter basis) of feed used in the study

Ingredients	Composition (g kg^−1^) dry feed
Cotton seed cake meal	683.40
Maize bran meal	316.60
Total	1000.00
*Proximate composition of feed*	
Dry matter	924.70
Crude protein	297.50
Crude fibre	136.70
Crude fat	109.60
Ash	68.10
Carbohydrate	388.10

### Determination of gonad maturity stages

Ten *O. niloticus* females were collected using a small seine net (12.7 mm) and were sacrificed by immersing them in an ice-slurry before being dissected for reproductive studies. The gonads were carefully excised, trimmed to remove extraneous fat and connective tissue in order to determine the maturity stage of the gonads. The maturity stages of the fish gonads were determined through visual inspection of the appearance, size and texture, according to the scale adopted from Mous et al. (1995) as shown in Table 2.

**Table 2:** Description of maturity stages of female *O. niloticus* as adopted from Mous et al. (1995)

*Stage I:* Immature: Appearance like testes; sexes indistinguishable macroscopically
*Stage II:* Early developing: Ovaries to be recognized by small whitish dots (eggs); caudal part of the ovaries more thickened than the rostral part
*Stage III:* Developing or recovering: Eggs developing inside the ovaries unequal in size
*Stage IV:* Early ripening: Eggs equal in size but not fully grown; all coloured yellow
*Stage V:* Ripe: Eggs large and ovaries visible from the ventral side of the cavity
*Stage VI:* Spent: Eggs or juveniles in the buccal cavity; ovaries recovering, thin and reddish; eggs unequal in size, often including a few residual stage V eggs

### Determination of size at first maturity (L_50_)

The L_50_ is the length of the onset of sexual maturity, i.e. stage IV (Marriot et al. 1997) at which 50% of females have reached sexual maturity. For this purpose, females were grouped into immature (stages I–III) that are not ready to spawn in the nearest breeding cycle and mature females (stages IV and V) that are going to spawn in the next spawning cycle. The L_50_ was estimated using the following equation (Silberberg et al. 2001):

$$P\; = \;\frac{1}{{1\; + \;e^{ - a(L - b)} }}$$

where *P* = proportion of mature fish at a specific length class (measured as total length); *a* and *b* are model parameters to be estimated; *L* = total length.

### Determination of condition factor

The condition factor (*K*) is used to compare the condition (‘fatness’) and the wellbeing of a fish and according to Bagenal (1978) heavier fish at a given length are in better condition. The condition factor (*K*) was calculated as:

$$K\; = \;\frac{100w}{{l^{3} }}$$

where *w* and *l* are the individual fish weight (g) and total length (cm), respectively.

### Estimation of gonado-somatic index (GSI)

Gonado-somatic index (GSI) is a measure that describes the state of maturity of a fish by expressing the weight of the gonads as a percentage of fish weight. Normally, the value of GSI increases as the development of fish gonads approaches ripeness; the value begins to decrease as the fish starts to spawn. The GSI was calculated using the following formula:

$${\text{GSI}}\; = \;\frac{{{\text{Gonad}}\;{\text{weight}}}}{{{\text{Weight}}\;{\text{of}}\;{\text{evicerated}}\;{\text{fish}}}} \times \;100$$

### Determination of fecundity and egg diameter

Fecundity is estimated from the gonads in the final maturation stage by counting oocytes having the largest diameter (Duponchellé et al. 2000). The absolute fecundity is the total number of ripe eggs prior to the next spawning period. The relative fecundity is the total number of ripe eggs per gram of female body weight (Bagenal 1978). Absolute fecundity was determined by counting the number of eggs in the ripe gonads through a gravimetric sub-sampling method (Bagenal 1978). The procedure involved weighing of the whole ripe gonads (stage V) stored in 4% formaldehyde. A small portion from each lobe taken from the posterior, middle and anterior regions was weighed and the number and weight of ripe eggs were determined. The total number of ripe eggs in the gonad from a single fish was calculated by using the following formula:

$$E\; = \;\frac{{\mathop w\nolimits_{g} \;\mathop e\nolimits_{s} }}{{\mathop w\nolimits_{e} }}$$

where *E* is the total number of eggs in a gonad; *w*_*g*_ is weight of the gonad; *e*_*s*_ is number of eggs in the sample; and *w*_*e*_ is weight of eggs in the egg sample.

The most advanced maturity stage encountered was determined by counting ovaries with GSI larger than 3% in order to determine the absolute fecundity (de Graaf et al. 1999). The egg size was determined by measuring the diameter of 40–55 randomly selected eggs per female along two axes in the counting chamber under a microscope using a calibrated eyepiece ocular micrometer at 40× magnification. At the end of the study, a comparison was made in terms of growth and yield performance between monoculture and polyculture systems as done by Shoko et al. (2011).

### Statistical analyses

Data were tested for homogeneity of variances using Levene’s test. A two tailed *t* test was used to compare the estimates of L_50_, fecundity, condition factor, GSI, growth and yield between monoculture and polyculture systems. Linear regression analysis was used to test the relationship between fecundity and total length; fecundity and total weight and fecundity and oocytes diameter in monoculture and polyculture systems. Linear regression analysis was also used to test the relationship between condition factor and absolute fecundity. Fisher’s Z-test was used to compare correlation coefficients (r) between the two culture systems (Zar 2010). All statistical analyses were performed using SPSS 13 for Windows (Landau and Everit 2004). Significance of differences was judged at p < 0.05.

## Results

**Figure 2:**
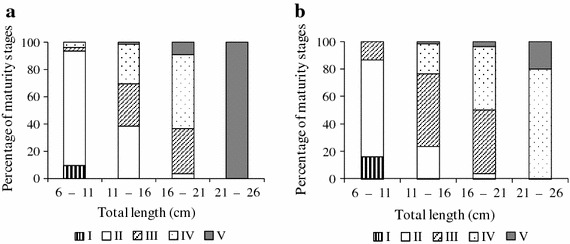
Percentage of each maturity stage for 5 cm length groups of female *O. niloticus* reared under monoculture (**a**) and polyculture (**b**).

Mature gonads of female *O. niloticus* reared under monoculture and polyculture systems started to appear at total length of 6–11 cm and 11–16 cm, respectively (Figure 2a, b). In both culture systems, the percentage of mature females increased with the increase in female length.

**Figure 3:**
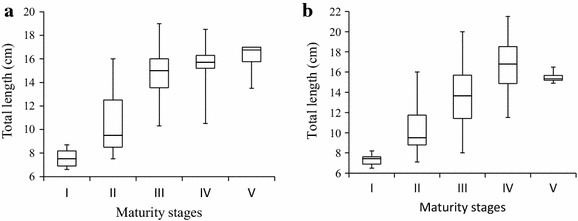
*Box-*and*-whisker plots* showing the total lengths (cm) of *O. niloticus* females within each maturity stage reared under monoculture (**a**) and polyculture (**b**) conditions.

Overlaps in total lengths among the five maturity stages from both culture systems were observed as indicated by the error bars in Figure 3 based on medians and percentiles for each category. The immature stages were observed in small females in both systems (Figure 3a, b).

**Figure 4:**
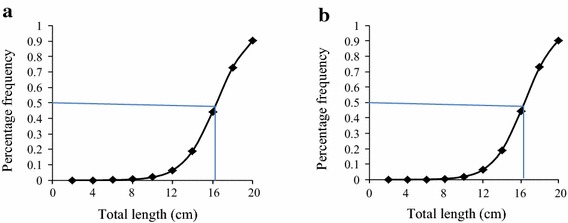
Length at L_50_ sexual maturity of female *O. niloticus* reared under monoculture (**a**) and polyculture (**b**) conditions.

Females reared under monoculture and polyculture systems attained the L_50_ at a total length of 16.38 and 16.59 cm, respectively (Figure 4a, b) with no significant difference (t = 1.96, df = ∞, p > 0.05) between the two culture systems.

**Figure 5:**
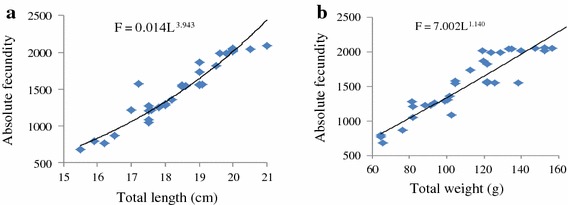
Relationship between absolute fecundity and total length (**a**), absolute fecundity and total weight (**b**) reared under monoculture system.

**Figure 6:**
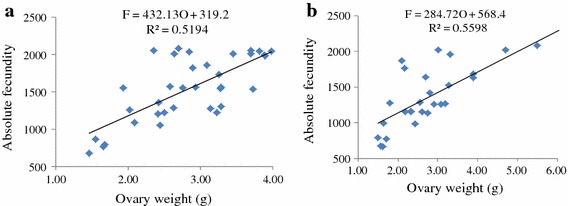
Relationship between absolute fecundity and ovary weight of *O. niloticus* reared under monoculture (**a**) and polyculture (**b**) system.

**Figure 7:**
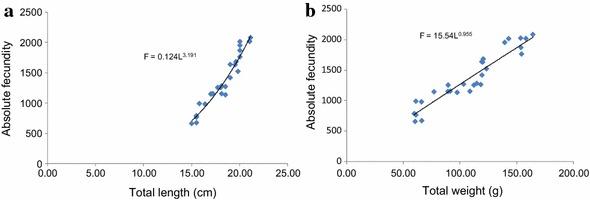
Relationship between absolute fecundity and total length (**a**), absolute fecundity and total weight (**b**) reared under polyculture system.

The absolute fecundity of *O. niloticus* reared in monoculture and polyculture ponds ranged from 678.00 to 2,086.00 and 662.00–2,096.00 with the corresponding total lengths of 15.50–21.00 and 15.00–21.20 cm, respectively. The absolute fecundity of females reared in monoculture was strongly correlated with total length (r = 0.96, df = 33, p = 0.000) and total weight (r = 0.92, df = 33, p = 0.000) (Figure 5a, b) while it was weakly correlated with ovary weight (r = 0.72, df = 33, p = 0.000) (Figure 6a). Likewise, absolute fecundity was also strongly correlated with total length (r = 0.91, df 31, p = 0.000) and total weight (r = 0.89, df = 31, p = 0.000) (Figure 7a, b) than with ovary weight (r = 0.72, df = 31, p = 0.000) in polyculture system (Figure 6b). However, there was no significant difference in absolute fecundity between the two systems (t = 1.810, df = 66, p = 0.075).

**Figure 8:**
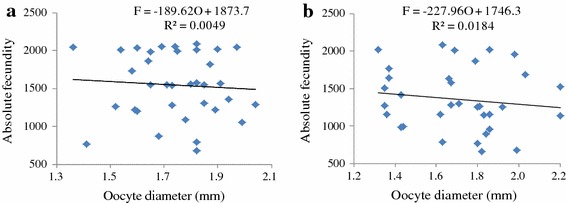
Relationship between absolute fecundity and oocyte length of *O. niloticus* reared under monoculture (**a**) and polyculture (**b**) system.

Oocyte diameter ranged from 1.36 to 2.04 mm and 1.32–2.20 mm in monoculture and polyculture, respectively. No significant correlation existed between absolute fecundity and oocyte diameter in monoculture system (r = 0.07, df = 33, p = 0.0688) (Figure 8a). Similarly, *O. niloticus* cultured in polyculture system did not show any significant correlation between absolute fecundity and oocyte diameter (r = 0.13, df = 31, p = 0.452) (Figure 8b).

No significant difference was observed in GSI (t = −1.012, df = 66, p = 0.315) and condition factor (t = 0.172, df = 66, p = 0.864) between the two culture systems with no relationship between the two parameters. However, a negative relationship between condition factor and relative fecundity under both culture systems was realized.

There were no significant differences in correlation coefficients of fecundity and total length (z = 1.748, p = 0.941), total weight (z = 0.681, p = 0.592) and oocytes diameter (z = 0.263, p > 0.257) between monoculture and polyculture systems. *O. niloticus* reared under monoculture showed significantly lower mean specific growth rates (t = 2.669, df = 178, p = 0.008), total weight gain (t = −5.277, df = 1,782, p = 0.000) and individual final weight (t = −5.217, df = 162, p = 0.000) than in polyculture (Table 3).

**Table 3:** Mean growth rates and yield parameters of *O. niloticus* cultured under monoculture and polyculture ponds (data are mean ± SE)

Production parameter	Monoculture	Polyculture
Average initial weight (g)	5.01 ± 0.05^a^	5.01 ± 0.05^a^
Rearing period (days)	240	240
Average final weight (g)	123.64 ± 2.57^a^	146.58 ± 3.56^b^
Total weight gain	118.63 ± 2.58^a^	141.57 ± 3.56^b^
Survival rate (%)	92.13 ± 1.70^a^	96.62 ± 3.97^b^
Specific growth rates (%)	1.33 ± 0.01^a^	1.40 ± 0.04^b^
Gross fish yield (tonha^−1^)	10.27 ± 0.55^a^	15.25 ± 0.64^b^
Net fish yield (tonha^−1^)	10.26 ± 0.54^a^	15.25 ± 0.64^b^
Net annual yield (tonha^−1^yr^−1^)	1797.25 ± 95.44^a^	2671.30 ± 111.55^b^

Values with dissimilar superscripts in a row are significantly different (*t* test).

## Discussion

The present study was conducted to determine the reproductive biology of female *O. niloticus* reared in monoculture and polyculture pond systems. The results from this study negate the hypothesis that *O. niloticus* reared in monoculture would attain sexual maturity earlier and at a smaller size than those in polyculture. This indicates that early breeding in *O. niloticus* under captivity is not affected by the type of culture system used.

Findings of this study are in agreement with those reported by de Graaf et al. (1999) who suggested that pond environmental conditions are not a major cause for precocious breeding of Nile tilapia. However, Lowe-McConnell (1982) stated that early breeding in *O. niloticus* can be influenced by the low condition factor which is influenced by the quality of food and protein levels (Gunasekera et al. 1995). In the present study, 50.00% of females reached sexual maturity at body length of 16.38 and 16.59 cm in monoculture and polyculture, respectively. These values are comparable to those reported by Peña-Mendoza et al. (2005), but higher than those (8.00–13.00 cm TL) reported by de Silva and Radampola (1990) and Siraj et al. (1983). More importantly, it seems that *O. niloticus* used in the present study exhibited neoteny (early breeding). However, neoteny did not reduce somatic growth because specific growth rates in both systems are similar to 1.35 and 1.47% obtained by Abdel-Hakim et al. (2008) from monosex rearing of Nile tilapia. Specific growth rates of 1.33 and 1.40% were obtained from monoculture and polyculture systems respectively in this study.

The present study showed overlaps in the ranges of total length among the different maturity stages. This is attributed to the fact that, maturation in *O. niloticus* does not depend only on length but it is also controlled by other factors such as food availability and water quality (Babiker and Ibrahimu 1979; Southgate and Lucas 2012). Changes in these factors are translated as neural signals which stimulate the pituitary gland and gonads to respond to the above triggers (Southgate and Lucas 2012).

The insignificant difference in GSI between monoculture (3.86%) and polyculture (4.33%) indicates that culture system has no effect on GSI. The GSI values from the present study are comparable with the maximum GSI of 3.60, 4.00, 4.70 and 4.80% reported by Babiker and Ibrahimu (1979), Hirpo (2012), Ambali and Little (1996) and Behrends and Smitherman (1983), respectively but lower than the 7.00% reported by de Graaf et al. (1999). The difference in experimental conditions could have contributed to the differences in maximum GSI obtained from the present study.

The insignificant difference obtained in the absolute fecundity of *O. niloticus* raised in monoculture and polyculture systems further support the notion that culture system has no effect on the reproductive capacity of the females. In the present study, the absolute fecundity ranges of 678.00–2,086.00 and 662.00–2,096.00 recorded in monoculture and polyculture, respectively are higher than the ranges of 289.00–1,456.00 and 104.00–709.00 for *O. niloticus* with 10.00–23.00 cm and 12.50–20.90 cm reported by Shalloof and Salama (2008) and Gómez-Márquez et al. (2003) respectively. Furthermore, the fecundity reported in the present study is lower than the maximum absolute fecundity of 2,603.00 with a corresponding total length of 34.90 cm reported by Peterson et al. (2004). Variation in fecundity could be attributed to the differences in experimental conditions.

In the present study absolute fecundity of *O. niloticus* reared under both monoculture and polyculture was strongly correlated with weight and total length than with ovary weight. These results are consistent with the results obtained by Gómez-Márquez et al. (2003), Murua et al. (2003), Peterson et al. (2004) and Shalloof and Salama (2008) who found that fecundity was directly correlated with body total length and weight. No correlation existed between absolute fecundity and oocyte diameter in *O. niloticus* cultured under either system. This is because in fishes, the size of ripe eggs does not vary much with the size or age of the fish. This finding is in agreement with that of Shalloof and Salama (2008). The size of oocytes reported from the present study ranged from 1.36 to 2.04 mm and 1.32–2.20 mm in *O. niloticus* reared under monoculture and polyculture ponds, respectively. These oocyte sizes are comparable with the size of 1.00–3.00 mm reported by Gómez-Márquez et al. (2003) and 1.80–2.45 mm reported by Babiker and Ibrahimu (1979), Siraj et al. (1983), Smitherman et al. (1988), de Graaf et al. (1999) and Shalloof and Salama (2008).

The significantly higher growth and yield of *O. niloticus* reared in polyculture than the monoculture system can be attributed to the reduced number of *O. niloticus* fingerlings as a result of predation by *C. gariepinus* and to the less competition for food and space. This improved growth performance in polyculture is in agreement with the findings reported by de Graaf et al. (1996), El Gamal et al. (1998), Liti et al. (2002) and Offem et al. (2009).

This study showed that, female size at first maturity in *O. niloticus* is not affected by the culture systems used. Moreover, adding *C. gariepinus* to Nile tilapia population reduced the number of unwanted tilapia fingerlings and improved growth performance and yield. However, further studies are necessary to determine the size of *C. gariepinus* for maximum elimination of the unwanted tilapia population for maximum tilapia growth and yield.
